# Visualising recalcitrance by colocalisation of cellulase, lignin and cellulose in pretreated pine biomass using fluorescence microscopy

**DOI:** 10.1038/srep44386

**Published:** 2017-03-10

**Authors:** Lloyd Donaldson, Alankar Vaidya

**Affiliations:** 1 Scion, 49 Sala Street, Private Bag 3020, Rotorua 3010, New Zealand

## Abstract

Mapping the location of bound cellulase enzymes provides information on the micro-scale distribution of amenable and recalcitrant sites in pretreated woody biomass for biofuel applications. The interaction of a fluorescently labelled cellulase enzyme cocktail with steam-exploded pine (SEW) was quantified using confocal microscopy. The spatial distribution of Dylight labelled cellulase was quantified relative to lignin (autofluorescence) and cellulose (Congo red staining) by measuring their colocalisation using Pearson correlations. Correlations were greater in cellulose-rich secondary cell walls compared to lignin-rich middle lamella but with significant variations among individual biomass particles. The distribution of cellulose in the pretreated biomass accounted for 30% of the variation in the distribution of enzyme after correcting for the correlation between lignin and cellulose. For the first time, colocalisation analysis was able to quantify the spatial distribution of amenable and recalcitrant sites in relation to the histochemistry of cellulose and lignin. This study will contribute to understanding the role of pretreatment in enzymatic hydrolysis of recalcitrant softwood biomass.

As world economies become more averse to fossil fuel utilisation due to declining supply and increases in atmospheric CO_2_ associated with climate change, there is increasing interest in woody biomass as a significant resource for the production of sustainable liquid fuels and biochemical feedstocks^1^,^2^. Woody biomass requires chemical or physical pretreatment to increase accessibility to cellulolytic enzymes in order to achieve acceptable conversion yields to fermentable sugars^3^. Crude biomass is generally recalcitrant towards enzymatic conversion because the cellulose substrate is embedded in a dense lignin-hemicellulose matrix. A number of mechano-chemical and thermo-chemical pretreatments have been developed which decrease recalcitrance by a combination of reduced particle size and chemical or mechanical disruption of the nanoscale structure of cell walls, resulting in either lignin/hemicellulose removal or separation of lignin/hemicellulose and cellulosic components^4^,^5^,^6^. While the mechanism of pretreatments varies, the common goal is to increase accessibility of cellulose to cellulase enzymes by reducing particle size and increasing particle porosity.

In addition to pretreatment, the saccharification of both softwood and hardwood biomass can be improved by using various additives in small amounts. Polyethylene glycol (PEG) is a relatively low-cost additive which improves saccharification probably by inhibiting non-productive binding of cellulase enzymes to lignin within the biomass^7^.

A number of studies have focussed on nanoscale interactions between enzymes and model substrates such as purified cellulose using atomic force microscopy and fluorescence microscopy^8^,^9^,^10^,^11^,^12^. Microscopy techniques have been used to model pore evolution during digestion^13^,^14^, to show how enzyme interaction with cellulose is limited by congestion on the surface of crystallites and by surface roughness^15^,^16^,^17^, and that chemical treatments to reduce roughness and addition of supplementary enzymes such as cellobiohydrolase may lead to improved efficiency of hydrolysis^18^. Measurements of the relative adhesion forces between cellulase enzymes and cellulose or lignin using atomic force microscopy^19^ have indicated that cellulase had a 45% stronger adhesion force with lignin than with cellulose, thus accounting for non-productive binding to lignin which reduces saccharification efficiency.

Similar studies on pretreated biomass have concentrated on effects of pretreatment. Steam explosion, dilute acid and hydrothermal pretreatments result in lignin redistribution and removal of hemicellulose that improves porosity and increases exposure of cellulose substrate to enzymes^20^,^21^,^22^,^23^,^24^,^25^. Several studies have indicated that disruption of nanostructure has a stronger effect on saccharification yield than lignin removal or changes to cellulose crystallinity^26^,^27^. Cell wall dislocations in fibres have been shown to be strong binding locations for cellulase during saccharification with a fluorescently labelled enzyme^28^. Cellulase also showed a preference for parenchyma cell walls in hydrothermally pretreated wheat leaves and straw, and in this particular case, the abundance of parenchyma was more important than the disruptive effects of the pretreatment^29^. In hardwoods (wood from flowering trees), tension wood containing highly porous and unlignified cell wall layers may be much more amenable to saccharification^30^. Hardwoods are generally more amenable to saccharification than softwoods (wood from conifers) due to differences in hemicellulose and lignin composition that make pretreatments more effective^31^.

Fluorescence microscopy has been particularly useful to localise labelled enzymes and to characterise enzyme-substrate interactions within biomass^32^. Most research has investigated nanoscale interactions between enzyme and substrate whereas studies which have examined microscale distribution of labelled enzyme on pretreated biomass have relied on qualitative visualisation in relation to cell and tissue type^28^,^29^. Fluorescence colocalisation is a technique for quantifying the spatial colocalisation of two or more fluorescent markers^33^,^34^,^35^,^36^,^40^. The simplest approach is to show overlay images where green and red markers of similar intensity appear as yellow when colocalised^33^. Colocalisation may reflect underlying nanoscale interactions such as binding but positive colocalisation does not directly imply binding or other types of molecular interaction. Colocalisation can be quantified using Manders’ coefficients representing the fraction of the distribution of one marker that overlaps with another, taking a value between 0 and 1.

Pearson correlations can also be used to measure colocalisation based on the correlation of marker intensities, taking values from −1 to +1^41^. When combined with Förster resonance energy transfer (FRET), colocalisation can be further quantified by proximity^35^,^36^ and detection of bound and unbound states^42^.

In colocalisation using Pearson correlation, a pixel-wise comparison of two intensity signals is performed after applying a threshold to exclude empty areas of the image. A positive r value indicates a colocalisation between signal intensities, a 0 value indicates random variation between signal intensities, whereas a negative correlation indicates an exclusion relationship where signals are at different locations from each other^40^,^41^. In the latter case, Manders’ overlap coefficients will be close to 0.

Our aim was to use colocalisation analysis to quantify the distribution of bound cellulase enzymes relative to cell wall histochemistry within pretreated pine biomass and to directly visualise and quantify the location of amenable or recalcitrant sites as revealed by enzyme binding. This study will contribute to understanding the role of pretreatment in enzymatic hydrolysis of recalcitrant softwood biomass.

## Results

### Enzyme labelling & saccharification

The cellulase enzyme used in these experiments contained a range of cellobiohydrolases (CBHs) and endo-1,4-β-glucanases (EGs) as well as smaller amounts of other accessory enzymes^43^. While all of these proteins were susceptible to labelling^44^, only those with strong binding to cellulose surfaces, including Cel7A^45^, were available for microscopic detection after washing of the biomass. Our labelled enzyme, therefore, represents at least 2 different protein molecules with CBHI (Cel7A) dominating^43^.

The labelled enzyme contained 7.48 moles of dye per mole of protein. The labelled protein (0.7 μM) was diluted by 1:200 in water for use in imaging experiments. Both Dylight labelled enzyme and unbound Dylight showed similar fluorescence intensity after exposure to the optimum saccharification temperature of 50 °C for 24 h indicating stability of protein labelling. However, no labelled enzyme was detected on SEW after 24 h at 50°, probably due to cellulose hydrolysis as indicated by weak Congo red staining. Therefore experiments were performed at room temperature (22 °C) to optimise enzyme detection while limiting the amount of saccharification (Table 1). The yield of reducing sugars at 22 °C was about 10% of the yield at 50 °C. Labelled enzyme had a 30% reduction in the yield of reducing sugars relative to unlabelled enzyme at 50 °C but there was little difference at 22 °C (Table 1) so there was some evidence of stearic hindrance due to Dylight labelling^44^.

### Microscopy

**Figure 1:**
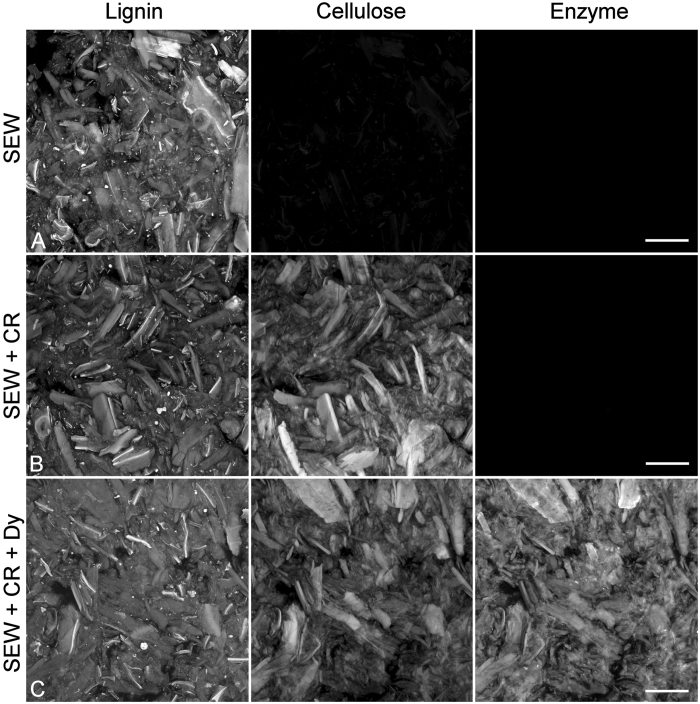
Control images for SEW (**A**), SEW + Congo red (CR, cellulose) (**B**), and SEW + Congo red + Dylight (Dy, enzyme) (**C**) at constant gain demonstrating the lack of bleed through as a result of sequential excitation. These images confirm that lignin autofluorescence does not contribute significantly to cellulose or enzyme detection due to the greater brightness of these components. Scale bar = 50 μm.

Sequential excitation enabled visualisation of lignin fluorescence, stained cellulose, and labelled enzyme without any significant bleed-through of signals apart from a very weak (<10 grey levels) contribution of lignin fluorescence to the cellulose channel (Fig. 1). Lignin fluorescence occurred at all wavelengths but declined with increasing excitation wavelength so to detect pure Congo red and Dylight signals these must be significantly brighter than the lignin fluorescence background as demonstrated in Fig. 1. In the case of purified lignin, autofluorescence was significantly increased by the purification treatment so that Dylight labelled enzyme could only be detected as a differential signal convolved with lignin autofluorescence. This had the effect of limiting sensitivity but unambiguous detection of Dylight was possible due to the poor correlation of Dylight intensity with lignin intensity (r = −0.21). To calculate Pearson correlations for purified lignin it was necessary to subtract the lignin emission measured at 500–570 nm from the combined lignin/Dylight emission measured at 650–750 nm. The correlation of lignin signals at the two emission wavelength ranges in the absence of Dylight was 0.96 which was sufficient to justify the image processing required to detect Dylight fluorescence.

Lignin autofluorescence appeared unaffected by either Congo red staining or the presence of Dylight labelled enzyme (Fig. 1). Congo red did not stain purified lignin. The fluorescence of Dylight labelled enzyme was slightly reduced in the presence of Congo red staining probably as a result of fluorescence quenching^42^. Congo-red staining was performed after labelled enzyme treatment in all experiments to avoid any interference with enzyme binding. FRET measurements indicated no detectable energy transfer between these fluorophores using acceptor photobleaching. This may indicate either a lack of colocalisation at the nanoscale between Congo red and labelled enzyme due to mutual size exclusion by competitive binding or the fluorophores may be constrained from achieving a close proximity by their co-alignment with the cellulose microfibrils or by the significant difference in size of the two molecules^42^.

**Figure 2:**
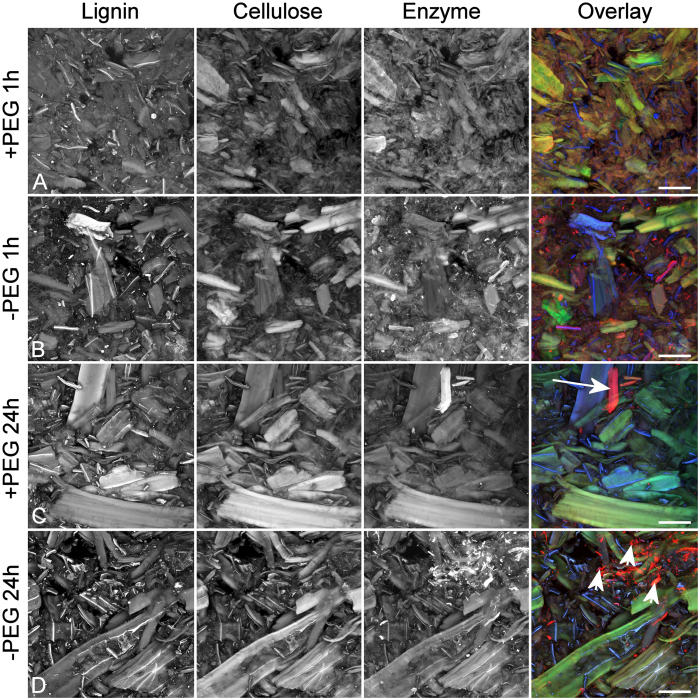
Component images for different combinations of PEG additive and time (**A**–**D**) and their overlay images demonstrate the heterogeneity of the pretreated biomass but with no obvious visual difference among treatments. Blue = lignin, green = cellulose, red = enzyme. Some highly amenable fibres with strong binding to labelled enzyme may be unlignified parenchyma cell walls originating from resin canals or rays (long arrow). Enzyme aggregates associated with fines are also present (short arrows). Scale bar = 50 μm.

Imaging and colour overlays of the three components, lignin (blue), cellulose (green), and enzyme (red), revealed a complex inter-relationship indicating that most individual fibre fragments had a different arrangement of the lignin, cellulose and enzyme signals as shown by a unique RGB (red, green, blue) colour in Fig. 2. Processing all three channels to have the same average brightness avoided the overlay image being dominated by the brightest signal, usually lignin, thus improving contrast among fibres with different signal combinations. Overlay images indicated that the SEW substrate was surprisingly heterogeneous (Fig. 2).

For SEW substrate, Manders’ coefficients between enzyme and cellulose were invariably 1 indicating that the distribution of enzyme overlaps completely with the detected cellulose and *vice versa*. These coefficients don’t provide any details on how the amount of bound enzyme is related to the amount of accessible cellulose. To quantify this relationship between accessible cellulose and labelled enzyme we used Pearson correlations between signal intensities on the fibre.

### Correlations–enzyme, cellulose, lignin

Individual r-values based on images have high degrees of freedom because of the large number of pixel comparisons, leading to small confidence limits for individual r-values. For the current experiment, individual correlation coefficients had a confidence interval of ±0.001 with an average number of degrees of freedom of 880,846. Using this confidence interval to test experimental effects is inappropriate and only indicates that individual r-values are accurate. Likewise, using series of optical sections as replicates is unlikely to represent random sampling as adjacent images in a series are likely to be similar. To test for differences among experimental treatments, replicated r-values offer a more realistic and conservative approach that satisfies the assumption of random sampling and we have used 6 replicates per treatment to assess variation with treatment time and the presence or absence of PEG additive. For general interpretation we consider r = 0 to indicate a random relationship between signals, r < 0.5 to indicate a low association, r = 0.5 to 0.8 to indicate a moderate association, and r > 0.8 to indicate a strong association.

**Figure 3:**
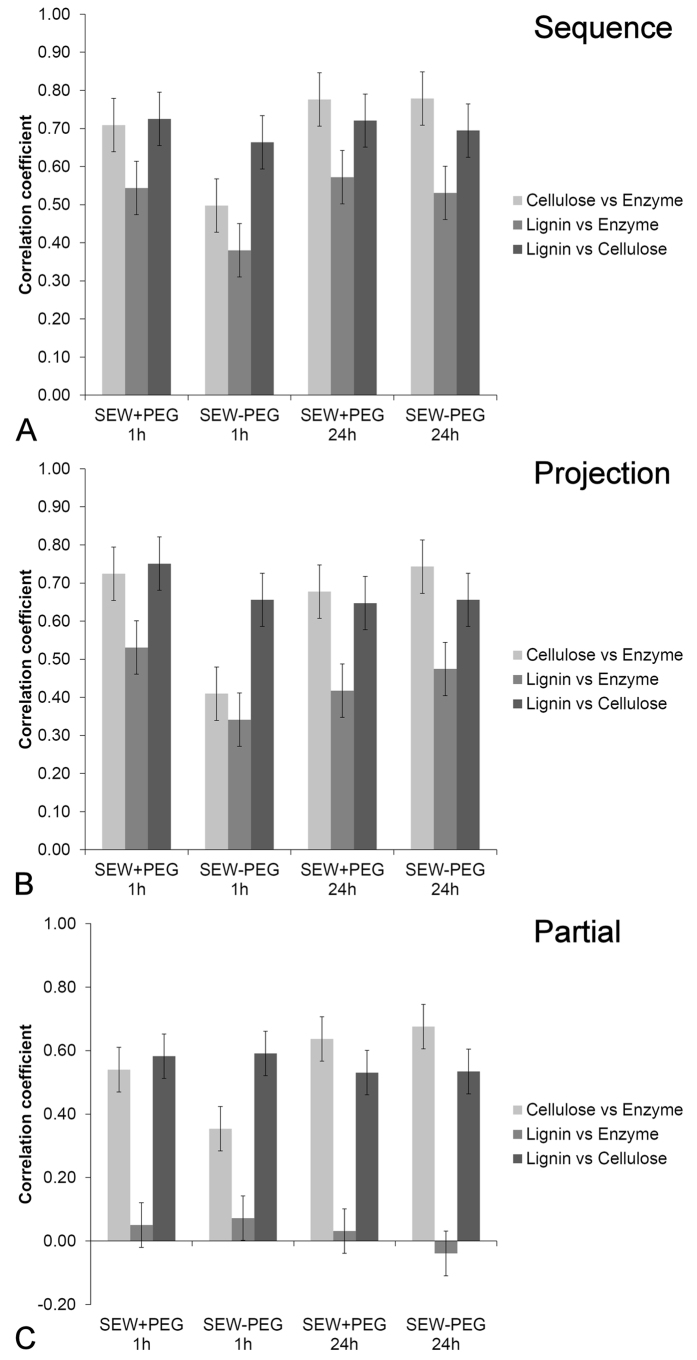
Average correlation coefficients demonstrate the difference in component associations with treatment time and presence or absence of PEG additive. The main experimental effect is the reduced correlation between cellulose and enzyme in the absence of PEG for the 1 h treatment. The correlation between lignin and cellulose remains constant among treatments. Projections (**A**) show similar trends to image sequences (**B**) whereas partial correlations (**C**) indicate that most of the lignin–enzyme association is accounted for by the cellulose–lignin association in secondary cell walls. Error bars represent 95% confidence intervals.

Measurement of correlations indicated some broad relationships (Fig. 3). Signals for cellulose and cellulase were moderately correlated as expected for an enzyme-substrate interaction (average r = 0.69). Enzyme treatment for 1 h in the presence of PEG gave a higher correlation (r = 0.71) compared to enzyme treatment without PEG (r = 0.50), whereas at 24 h there was no significant effect of PEG on the cellulose–enzyme correlation (r = 0.78) indicating the effect of PEG in improving cellulose–enzyme interaction was time dependent. Cellulose and lignin were also correlated since the cell wall contains both components (average r = 0.70) whereas lignin and enzyme were less correlated (average r = 0.51).

Correlations were found to be slightly greater when calculated on image sequences rather than projections (Fig. 3) as expected because projections may incorrectly colocalize objects that are separated in depth. However, the same general trends are shown by both sequences and projections indicating that this source of error is very small so projections could be used for the frequency scatter plots in order to simplify calculations.

For comparison, the correlation between images of SEW fibre containing only random intensities (created using image processing) with real intensity signals for lignin, cellulose and enzyme gave r values of approximately 0 (r = −0.03). The correlation between two independent random signals was also 0 (r = −0.01).

Calculation of partial correlations resulted in the lignin–enzyme correlation being reduced from 0.51 to 0.03 which suggests that the lignin–enzyme correlation may be due to the covariance of both with cellulose (Fig. 3). However, the lignin–enzyme correlation may also reflect non-productive binding of the enzyme to lignin in which case a random scatter of intensities would be expected to yield a value of r = 0. (For actual measurements see “Other substrates” below).

Analysis of variance performed on replicated partial correlation coefficients indicated the most significant main effect was the difference between the three components accounting for 95% of the variance. The remainder of the variance was accounted for by an interaction between component and time. For cellulose vs enzyme, the correlation increased with time. For the 1 h treatment, the cellulose–enzyme correlation was significantly reduced in the absence of PEG. The correlation between lignin and cellulose did not vary significantly. Coefficients of determination (r^2^) can indicate the proportion of the variation in enzyme distribution accounted for by the distribution of cellulose. The distribution of cellulose accounted for 30% of the variation in the distribution of enzyme after correcting for the correlation between lignin and cellulose (average r = 0.55). The remainder of the variation can be attributed to accessibility or perhaps to non-productive binding to lignin.

**Figure 4:**
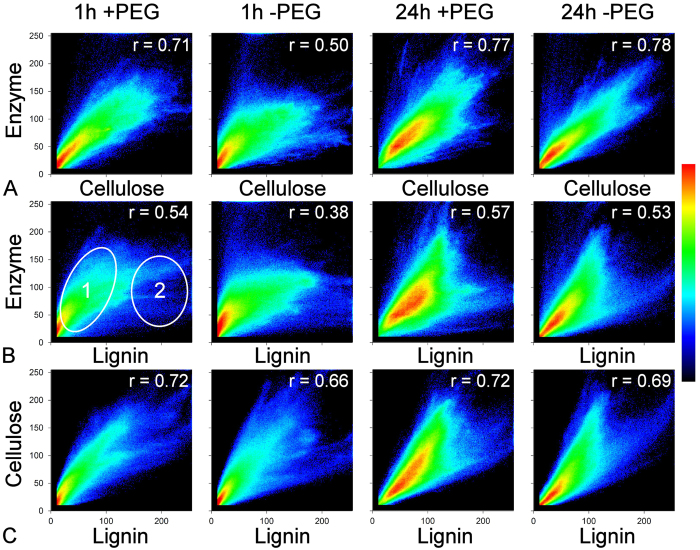
Average frequency scatter plots for lignin, cellulose and enzyme intensity demonstrate the pattern of component association with intensity. The enzyme shows a moderate linear correlation with cellulose (**A**) although this is notably weaker in the 1 h -PEG treatment. The scatterplots of enzyme vs lignin (**B**) show a more divergent pattern with low overall correlation. The correlation is reduced because there are two distinct associations. Low lignin points (1) represent secondary cell wall rich in cellulose and with higher affinity for the enzyme, while high lignin points (2) represent middle lamella with low cellulose and with weak affinity for the enzyme showing random scatter. The later may represent non-productive binding of the enzyme to lignin. Scatterplots show a moderate correlation of lignin and cellulose (**C**) which does not change with treatment. The intensity scale is 0–255 grey levels with an arbitrary frequency scale from lowest (black) to highest (red). Intensity has been thresholded at 10 grey levels.

Two-way frequency scatterplots of component interaction showed a moderate correlation between cellulose and enzyme (Fig. 4). For the enzyme–lignin interactions, scatterplots showed lower correlations. This reduced correlation was due to the presence of two distinct associations corresponding to different fibre types. Secondary wall with low lignin and high cellulose showed a stronger association with enzyme than more highly lignified fibre with less cellulose and hence a weak association with enzyme (Fig. 4). The reduced correlation also reflected the random scatter resulting from non-productive binding of the enzyme to lignin.

### Enzyme aggregation

**Figure 5:**
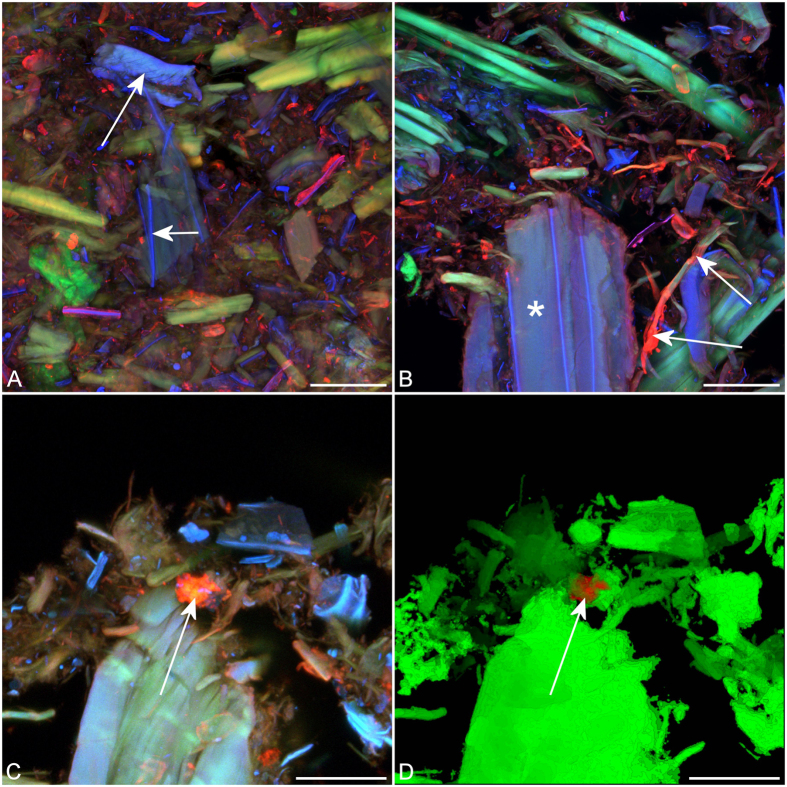
Overlay images show the distribution of enzyme on pretreated fibre. Example images showing details of colocalisation. Blue = lignin, green = cellulose, red = enzyme. (**A**) Blue structures (arrows) are highly lignified middle lamella fragments (short arrow) and a compression wood fibre (long arrow) identified by its increased lignin fluorescence and helical cavities (arrow). (**B**) Highly fibrillated fibre fragments show a strong affinity for labelled enzyme (arrows) while the highly lignified middle lamella forms a coating on some fibres with low enzyme affinity (asterisk). (**A**,**B**) Scale bar = 50 μm. (**C**) Maximum intensity projection of enzyme aggregation (arrow). (**D**) A surface rendered view of enzyme aggregation using depth shading. The surface rendered projection clearly shows the association between the enzyme aggregation and adjacent fines (arrow). (**C**,**D**) Scale bar = 20 μm.

**Figure 6:**
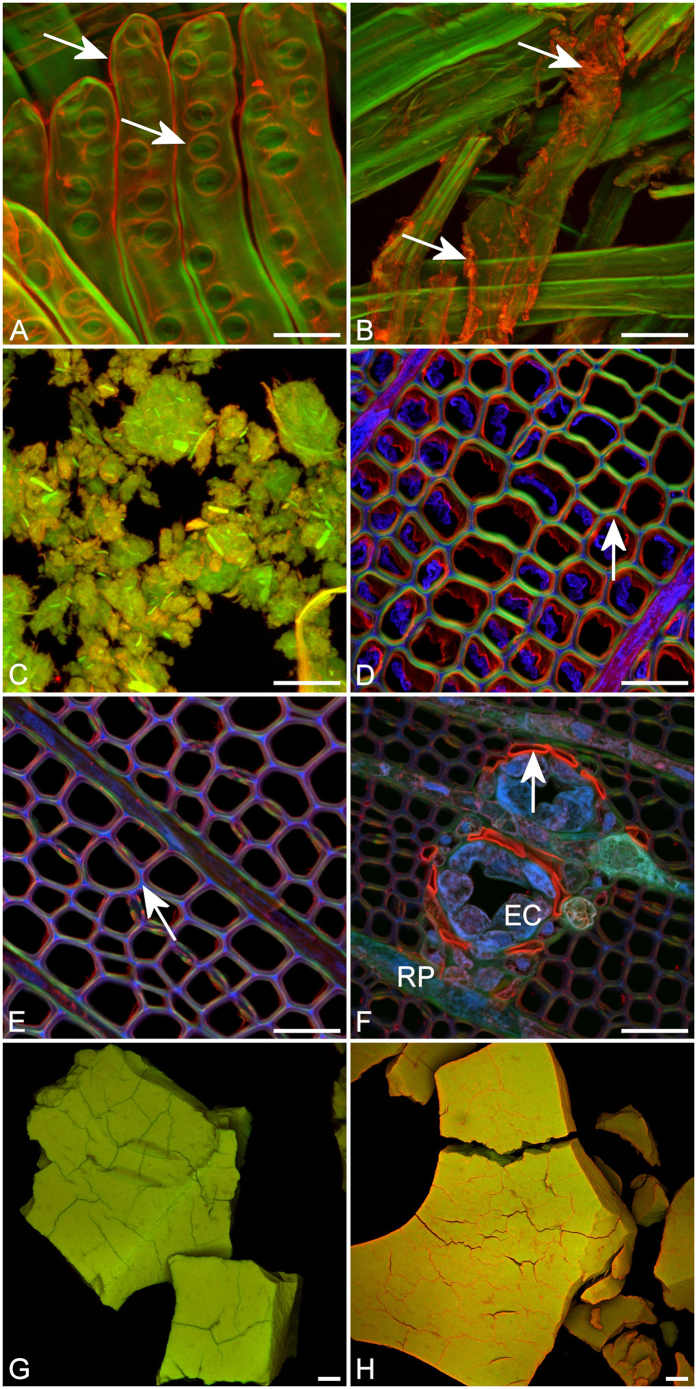
Comparison of labelled enzyme interactions with holocellulose, wood and lignin substrates demonstrates different types of component interactions. (**A**) In pine holocellulose, labelled enzyme (red) interacts strongly with the primary cell wall on the surface of tracheids especially at the pit border margins (arrows). (**B**) In SEW holocellulose, labelled enzyme interacts strongly with fines (arrows) but also infiltrates intact fibre walls. (**C**) In nanofibrillated cellulose, labelled enzyme interacts with most of the cellulose fibrils but infiltration is limited in larger particles. (**D**) In developing xylem, labelled enzyme interacts strongly with fibrillated cellulose on the lumen surface (arrow) but is excluded from most of the unlignified secondary cell wall (green) and lignified middle lamella (blue). The blue fluorescent material in the cell lumen is residual cytoplasm. (**E**) In wood sections, labelled enzyme shows a very weak interaction with the surface of the section in areas of cellulose-rich secondary cell wall but is excluded from the lignin-rich middle lamella (blue) (arrow). (**F**) Resin canal parenchyma cell walls show a strong affinity for labelled enzyme (arrow) in contrast to epithelial cell walls (EC) and ray parenchyma cell walls (RP). (**G**) Purified lignin autofluorescence. (**H**) Purified lignin (green) shows non-productive binding of labelled enzyme (red) on the surface and in cracks. Scale bars = 50 μm.

Most images contained discrete red objects representing an aggregation of the labelled enzyme. Close examination of images indicated that most of the smaller aggregates were associated with cell wall fragments or fines (Fig. 5). Since only some fines were associated with aggregates it seems likely that these fines may be fragments of unlignified cell wall from resin canal and ray parenchyma as indicated by their low level of autofluorescence. Treatment of transverse wood sections with labelled enzyme indicated that some unlignified parenchyma cell walls had much higher affinity for labelled enzyme than lignified secondary walls of tracheids (Fig. 6f).

### Other substrates

Comparison of SEW with pine holocellulose, SEW holocellulose, nanofibrillated cellulose, transverse sections of pine wood, and purified lignin, revealed a fundamental difference between these substrates with respect to their interaction with the labelled enzyme (Fig. 6). While SEW fibres were often fully infiltrated with Dylight labelled cellulase, pine holocellulose, wood, and purified lignin were labelled only on the surface of fibres, sections, or particles. Holocellulose made from SEW showed the same behaviour as SEW with most fibres and fines infiltrated with the labelled enzyme. The difference in behaviour of SEW holocellulose compared to pine holocellulose probably reflects the absence of hemicellulose which is removed during washing of the biomass after pretreatment^21^. Measurement of Pearson correlations for SEW holocellulose labelled with Congo red and Dylight labelled enzyme (lignin fluorescence was absent) indicated similar values to SEW (average r = 0.54 cf. SEW 1 h -PEG r = 0.50). Pine holocellulose showed labelling on the primary wall exposed on the outer surface of tracheids with only weak infiltration of the secondary wall (Fig. 6a).

Nanofibrillated cellulose showed the best infiltration (average r = 0.83) and this value is probably very close to the highest possible correlation between Congo red and Dylight labelled enzyme (Fig. 6c). Using dynamic light scattering we measured the hydrodynamic radius of Dylight labelled enzyme as 3.1 nm. Congo red is known to occur in aqueous solution as small oligomers with a hydrodynamic radius of 1.4 nm but the smaller monomeric form is likely to be more abundant in the ethanolic staining solution used in our experiments^46^. Since these two probes are very different in size their interaction with cellulose may not be exactly comparable although the size difference is very useful in terms of interpreting the effect of accessibility. Therefore, it is unlikely that the Congo red labelled cellulose and the Dylight labelled enzyme bound to cellulose will ever show an absolute correlation (r = 1). Congo red interacts with cellulose by hydrophobic interaction between phenolic groups and OH groups on cellulose^47^. Cellulase enzymes such as Cel7A interact with cellulose via a binding domain and a hydrolytic domain connected by a flexible linker but the exact mechanism of binding is still not fully understood^45^,^48^.

Dylight labelled cellulase bound strongly to the inner part of unlignified secondary cell walls in developing xylem (Fig. 6d) but not to the adjacent unlignified secondary wall in the outer part of the cell wall. This requires further detailed study but probably reflects the denser hemicellulose matrix in the outer cell wall of these developing tracheids.

Wood sections were labelled with enzyme on the transverse surface, and by Congo red throughout the section, reflecting the difference in the size of the two molecules (average r = 0.41; surface based on a projection r = 0.72) (Fig. 6e). Surface labelling was observed predominantly over the secondary cell wall with exclusion over the middle lamella region (Fig. 6e). Strong enzyme labelling of some resin canal parenchyma cells was also observed (Fig. 6f).

Purified pine lignin showed a thin coating of labelled enzyme on the exposed outer surface of the particles and to a limited extent within cracks on the surface (Fig. 6g,h). Measurement of Pearson correlations between lignin and Dylight labelled enzyme gave low negative correlations (average r = −0.21) reflecting both the surface location of the enzyme (exclusion) and the non-productive (random) nature of the association between lignin and enzyme.

## Discussion

The process of biomass deconstruction is well understood from a chemistry perspective but there have been relatively few studies examining microscopic aspects of pretreated biomass. There have been excellent studies examining how cellulase enzymes interact with purified cellulose in an effort to understand the molecular basis for substrate binding and hydrolysis^8^,^9^,^10^,^11^,^12^,^13^,^14^,^15^,^16^,^17^,^18^. However woody biomass is much more complex than purified cellulose. Depending on the pretreatment, biomass may contain varying amounts of hemicellulose and lignin. Particle size may vary from intact fibres to small fines, and the composition of fibre may vary with tissue type (softwood vs hardwood, early vs latewood, juvenile vs mature wood, and normal vs reaction wood). Since cellulase enzymes are invisible in microscopic preparations it is essential to either label the enzyme molecules with a fluorescent dye^44^ or to develop a highly specific probe such as an antibody that can be used for localisation of specific enzyme types such as cellobiohydrolase or endoglucanase. Several studies have used fluorescently labelled enzyme to perform a qualitative analysis of enzyme distribution on agricultural biomass^28^,^29^. In hydrothermally pretreated wheat straw, the importance of enzyme binding to cell wall dislocations in the early stages of saccharification was demonstrated^28^. In a comparison of pretreated wheat leaves and stems, enzyme binding to parenchyma cell walls was found to be an important predictor of digestibility^29^. In SEW, dislocations and some parenchyma cells were also observed to be strong binding sites for labelled enzyme (Figs 5 and 6).

In our study, we have taken this approach further in an attempt to quantitatively measure how labelled enzyme interacts with pretreated softwood fibre. We have determined to what extent the histochemistry of the fibre cell wall determines the distribution of bound enzyme. Measurement of the Pearson correlation between images of cellulose distribution and enzyme distribution indicates that only 30% of the variation in enzyme distribution can be explained by the intensity of cellulose staining with the remainder probably due to differences in accessibility. Treatments comparing presence and absence of PEG additive at two different treatment times indicates that for a 1 h treatment with the labelled enzyme, PEG additive improves the correlation of cellulose and enzyme from 0.5 to 0.7. Longer treatment time of 24 h improves the correlation further to 0.8 but the effect of PEG additive is no longer present. This quantitative assessment provides information that is not apparent from just looking at the images. The correlation between lignin and enzyme after correction for the correlation between lignin and cellulose is 0 indicating a random association which would be expected for non-productive binding.

Under ideal conditions, the Pearson correlation between cellulose and enzyme might be expected to approximate 1 but the highest value we observed was 0.83 for nanofibrillated cellulose. Because Congo red and Dylight labelled enzyme interact with cellulose in different ways and the two molecules are very different in size, images of the two signals are unlikely to ever be exactly the same. The similarity between the two signals appears to be reduced primarily because labelled enzyme shows a strong preference for interacting with biomass surfaces due to its larger size while the much smaller Congo red dye readily infiltrates biomass particles based on observations on pine holocellulose and wood sections. However, this surface binding effect appears to be much less important in the highly porous SEW substrate where significant infiltration of biomass particles by the labelled enzyme was observed in image sequences. Despite delignification, the distribution of labelled enzyme was largely restricted to the surface of pine holocellulose. Delignification of SEW substrate to produce SEW holocellulose did not change accessibility to the labelled enzyme as measured by Pearson correlation. These results confirm that accessibility is a major determinant of enzyme interaction compared to the composition of the substrate although the composition may have other effects on the rate of enzymatic saccharification^26^. Previous studies examining holocellulose saccharification have found that enzyme interacts mainly with fibre surfaces during early stages of saccharification but as hydrolysis progresses and porosity is increased, cellulose degradation occurs via infiltration^49^ rather than by surface erosion^48^.

Hemicellulose is also known to interfere with saccharification by masking cellulose to which it can hydrogen bond^50^. Our observations comparing pine holocellulose, which has a significant galactoglucomannan content, with SEW and SEW holocellulose where the galactoglucomannan has been removed by pretreatment and washing^21^, suggest a shielding effect. In pine holocellulose, the enzyme can only access cellulose on the fibre surface while in SEW and SEW holocellulose enzyme can infiltrate cell walls and thus access cellulose in the interior. Hot water extraction is a common pretreatment used to remove xylan from hardwood biomass^51^ and improve its saccharification efficiency but is less effective for removal of galactoglucomannans in softwoods^52^.

Variability among individual SEW fibres as shown by the colour variation in overlay images (Fig. 2) suggests that the pretreatment varies in its effectiveness on a micro scale indicating some scope for future improvements in such pretreatments. The Pearson correlation of cellulose and enzyme could, therefore, be a measurement of pretreatment effectiveness, a high value of r indicating high accessibility and a low value indicating recalcitrance. Simply measuring the saccharification yield does not provide information on the heterogeneity of the sample with respect to accessibility. The overlay images in Fig. 2 and Fig. 5 effectively show these features for individual components of the biomass. Other examples of apparent fibre heterogeneity are seen where some individual fibres show little or no affinity for labelled enzyme despite having strong Congo red staining. Some of these fibres show features consistent with compression wood (reaction wood associated with stem lean) (Fig. 5) such as helical checking and increased lignification suggesting that compression wood shows different behaviour from normal wood, as does tension wood in hardwood biomass^6^,^30^. Compression wood can be a significant component of softwood biomass varying among plantation sites as well as within trees so understanding its recalcitrance properties would be important in optimising any potential commercial process for biofuel production from softwood.

Aggregations of labelled enzyme were observed in all sample treatments but not in all images within each treatment. These distinctly red objects in overlay images were often associated with fines. Red objects were absent in control samples not treated with the labelled enzyme and in pine holocellulose where fines were absent. The red particles associated with fines probably represent enzyme binding to fragments of unlignified parenchyma cell walls from resin canals and ray cells due to exposure of cellulose. Some parenchyma cells showed a strong affinity for labelled enzyme when sections of untreated wood were exposed to labelled enzyme (Fig. 6f). Some larger particles represent large parenchyma cell fragments (Fig. 2). Preferential binding of labelled enzyme to parenchyma cell walls has also been reported for wheat straw^29^.

Using highly purified pine lignin, non-productive binding of cellulase enzyme to the surface of lignin particles was clearly demonstrated (Fig. 6). As far as we are aware this is the first time such interaction has been directly observed microscopically. However such non-productive interaction was not apparent in SEW containing highly lignified particles derived from middle lamella, nor was it observed on the surface of wood sections where highly lignified regions excluded labelled enzyme. Because lignin and cellulose are so intimately mixed at the nanoscale, distinguishing productive interaction with cellulose and non-productive interaction with lignin is difficult on mixed substrates. Since cellulose and lignin nanostructures are separated on a scale of about 3 nm even FRET cannot clearly distinguish the two types of association in a lignocellulosic substrate.

Lignin is thought to interfere with cellulase activity in two ways, masking of crystalline cellulose which is normally embedded in a lignin/hemicellulose matrix, and non-productive enzyme binding to lignin^53^,^54^. In SEW substrate, lignin and cellulose are spatially separated at the nanoscale with lignin particles suspended in an open web of cellulose^20^. In this type of pretreated substrate, non-productive binding of cellulase enzyme to lignin is likely to be the dominant method of interference. We found that removal of lignin from SEW did not change the colocalisation of cellulose and enzyme. Although we were able to directly observe non-productive binding of the enzyme to purified lignin, visualising this interaction in a mixed substrate remains a challenge.

## Conclusions

The distribution of labelled cellulase enzyme on steam-exploded pine fibre reveals a moderate correlation to cell wall histochemistry, especially with cellulose. Colocalisation analysis is a useful tool to quantify the distribution of labelled enzyme on pretreated biomass and demonstrates variable accessibility among pretreated fibres. This approach can be used to detect recalcitrant fibres and thus identify biomass components that may limit yield. Overall our study demonstrates that accessibility of pretreated biomass is the key factor in determining the distribution of enzyme with the distribution of lignin and cellulose playing a smaller role. We demonstrate that PEG additive improves the colocalisation between cellulose and enzyme within fibre walls. We demonstrate that delignification alone does not allow the enzyme to infiltrate fibre walls but the removal of galactoglucomannan by steam explosion does allow infiltration thus accounting for the effectiveness of this pretreatment. We were able to directly visualise non-productive binding of labelled enzyme with purified lignin for the first time.

## Materials and Methods

### Enzyme labelling

A cellulase cocktail from *Trichoderma reesei* ATCC 26921(C8546, lot # 031M1283V–Sigma-Aldrich Co. St. Louis MO63103, USA) was labelled with Dylight 633 (Thermo Fisher Scientific Ltd, Rockford, IL 61101, USA–Lot # NI 176327)^28^,^55^. 8.4 Milligrams of freeze dried cellulase powder was dissolved in 1 mL of 0.05 M borate-phosphate buffered saline at pH 7.5. This enzyme solution was mixed with Dylight reagent and vortexed gently to ensure complete mixing. The tube was covered with aluminium foil and incubated at 25 °C for 1 h with shaking at 50 rpm. The unlabelled dye in the reaction mixture was separated on 250 μl of supplied resin. The purified labelled protein was stored at 4 °C protected from light. Based on the reported average molecular weight (65 kDa) of cellulase (Cel7A and Cel7B) from *T. reesei*^44^ the molar ratio of Dylight to protein was found to be 7.48 M label per mole of protein. We did not investigate the relative labelling of the component enzymes in the cocktail as it is beyond the current scope to investigate differences in behaviour between the various enzymes present^44^.

The hydrodynamic radius of Dylight labelled cellulase was measured by dynamic light scattering.

### Saccharification

Unlabelled enzyme was prepared by dissolving 8.4 mg/ml of Cellulase powder from Trichoderma reesei ATCC 26921(C8546, lot # 031M1283V–Sigma-Aldrich Co. St. Louis MO63103, USA) into 50 mM Na-citrate buffer pH 4.8. The same concentration was used for the preparation of labelled enzyme. Both stock solutions were diluted 1:160 in citrate buffer. Saccharification was performed in the absence of PEG additive by adding 0.29 g of triple washed SEW substrate to 975 μl of 50 mM Na-citrate buffer pH 4.8. To this, 25 μl of either labelled or unlabelled enzyme was added giving a final dilution of 1:200, the same dilution as used for microscopy. The reaction mixture was incubated at 22° or 50 °C for 24 h at 180 rpm in an inclined position in an incubator shaker. A control saccharification experiment was performed using a corresponding amount of de-activated enzyme solution in the saccharification mixture, prepared by incubating the enzyme solution in a boiling water bath for 5 min and then cooling down to room temperature. The enzymatic saccharification was stopped by plunging the tube into a boiling water bath for 5 min and then cooling in water to bring it to room temperature. The mixture was then centrifuged at 6000 rpm for 10 min at 25 °C. The degree of enzymatic conversion was determined by measuring the amount of reducing sugars released in mg/L using the DNS (dinitrosalicylic acid) method^56^. The net reducing sugar released was calculated after subtracting the control measurements from the sample measurements.

### Substrate treatment

The optimal dilution of labelled enzyme for microscopy was found to be 1:200 from an initial concentration of 0.7 μM labelled protein. Steam-exploded radiata pine substrate^21^ was treated with excess labelled enzyme as described above with or without 0.1% w/w PEG (polyethylene glycol) for 1 h or for 24 h at room temperature (22 °C). Treated substrate was washed three times with distilled water, stained with 0.01% Congo red in 50% ethanol for 10 mins followed by a further water wash, and mounted in 50% glycerol in phosphate buffer at pH7. SEW was imaged using a Leica SP5 II confocal microscope using 488 nm, 561 nm, and 633 nm sequential excitation, and 500–570 nm (lignin autofluorescence), 570–620 nm (Congo red–cellulose), and 650–750 nm (Dylight–enzyme) fluorescence emission. A 20 × 0.7 NA glycerol immersion objective was used for imaging with 3x digital zoom. Controls were performed with and without Congo red, as well as with and without labelled enzyme. Image sequences for image correlation analysis were acquired using 50 optical slices at a step size of 1–2 μm. Six image sequences were acquired for each of the four treatments for analysis. Maximum intensity projections were made for illustrative purposes.

Pine holocellulose and SEW holocellulose were prepared by treating wood or SEW with peracetic acid (50:50 glacial acetic acid and 30% w/w hydrogen peroxide) at 90 °C for several hours until fully bleached. Sections of pine wood were prepared using a sledge microtome at a thickness of 30 μm. Nanofibrillated cellulose (BioPlus hydrophilic fibril gel, America Process Inc) was dispersed in distilled water to form a dilute slurry. Purified lignin was prepared by ionic liquid extraction, ball milling and extensive enzymatic digestion to completely remove polysaccharides^6^. Cellulose and lignin substrates were then treated with Dylight labelled cellulase for 1 h at room temperature, washed 3 times with distilled water and then stained with Congo red for 10 mins followed by brief washing and mounting in glycerol medium. Lignin was prepared in the same way except that Congo red staining was omitted. Cellulose and lignin substrates were examined by confocal microscopy and Pearson correlations were calculated as described below. A separate sample of lignin with no enzyme treatment was prepared to test for Congo red staining.

### Image analysis

Image correlation analysis was performed on image sequences (x, y, z = 1024 × 1024 × 50). Each image in the sequence contained components for lignin autofluorescence^57^ (blue fluorescence), cellulose stained with Congo red^47^ (green fluorescence), and Dylight labelled cellulase enzyme^28^ (red fluorescence). The Pearson correlation with a background threshold of 10 grey levels^41^ was calculated between pairs of components for each image in the sequence using V++ software (Digital Optics, Auckland, New Zealand). Manders’ coefficients were also calculated^37^,^38^. Confidence limits for individual Pearson correlations were calculated using the Fisher transform^58^. Average frequency scatterplots of correlations were prepared from maximum intensity projections of each sequence by summing individual data from the 6 replications. Correlation calculations were performed on raw data but scatterplots were rendered using normalised data to improve visualisation. Normalisation involved adjusting red, green and blue channels to have the same average intensity and the same dynamic range (0–255). Partial correlation coefficients were calculated, based on the raw sequence data^58^. Replicated correlation coefficient data based on 6 image sequences per treatment were analysed by analysis of variance^58^.

To compare Pearson correlations among actual data with random data a projection image of SEW fibre based on the logical OR of lignin and cellulose signals was binarised and the resulting fibre image filled with random intensities. Pearson correlations were then calculated between the random intensity image and each of the three real signals using maximum intensity projections. The Pearson correlation between two independently created random intensity images was also determined.

Enzyme aggregations were also studied by making surface rendered projections of enzyme and fibre using depth shading.

FRET measurements using acceptor photobleaching^35^,^36^,^42^ were performed using Dylight as the acceptor and Congo red as the donor.

## Additional Information

**How to cite this article:** Donaldson, L. and Vaidya, A. Visualising recalcitrance by colocalisation of cellulase, lignin and cellulose in pretreated pine biomass using fluorescence microscopy. *Sci. Rep.* **7**, 44386; doi: 10.1038/srep44386 (2017).

**Publisher's note:** Springer Nature remains neutral with regard to jurisdictional claims in published maps and institutional affiliations.

## Figures and Tables

**Table 1:** Saccharification measured as the yield of reducing sugars (mg/L).

	22 °C	50 °C
Labelled enzyme	8.3 ± 0.2	88.6 ± 10.0
Unlabelled enzyme	8.7 ± 0.5	125.9 ± 8.5

Values represent a mean of 3 measurements ± 95% confidence interval. Analyses were performed using a 1:200 dilution of the enzyme without PEG additive.
